# Willingness to Receive COVID-19 Vaccination among Older Adults in Saudi Arabia: A Community-Based Survey

**DOI:** 10.3390/vaccines9111257

**Published:** 2021-10-31

**Authors:** Mohammed Khaled Al-Hanawi, Noor Alshareef, Rehab H. El-Sokkary

**Affiliations:** 1 Department of Health Services and Hospital Administration, Faculty of Economics and Administration, King Abdulaziz University, Jeddah 80200, Saudi Arabia; naalshareef@kau.edu.sa; 2 Health Economics Research Group, King Abdulaziz University, Jeddah 80200, Saudi Arabia; 3 Medical Microbiology and Immunology Department, Faculty of Medicine, Zagazig University, Zagazig 44519, Egypt; rehab_elsokkary@zu.edu.eg

**Keywords:** COVID-19, older adults, hesitancy, Saudi Arabia, vaccine

## Abstract

Identifying the factors driving vaccine hesitancy can improve vaccine attitudes and motivate individuals to have the recommended vaccinations. However, failure to address the issue directly, or worse, ignoring it, could deepen such concerns, resulting in lower vaccination rates, leading to elevated rates of illness and vaccine-preventable deaths among older adults. The aim of this study was to explore the rate of acceptance of the COVID-19 vaccine among older adults in Saudi Arabia, along with the associated predicting factors and reasons for hesitancy. This study extracted data from a cross-sectional online survey on the acceptability of COVID-19 vaccination in Saudi Arabia, which was conducted from 8 to 14 December 2020. The sample of the study included 488 older adults aged 50 and older. The major data analytic tools employed in the study were bivariate and multivariable regression analyses. Among the 488 participants, 214 (43.85%) reported willingness to accept the COVID-19 vaccine when available. Older men were more likely to be willing to be vaccinated (adjusted odds ratio (aOR): 2.277; 95% confidence interval (CI): 1.092–4.745) than older women. High levels of education were significantly associated with willingness to be vaccinated. Older adults who had previously refused any vaccine were less likely to take the COVID-19 vaccine (aOR: 0.358; 95% CI: 0.154–0.830). Those who expressed a high or very high level of concern related to becoming infected were more likely to accept the vaccine against COVID-19 (aOR: 4.437; 95% CI: 2.148–9.168). Adverse side effects (27.01%), and safety and efficacy concerns (22.63%) were the most commonly cited reasons for vaccine hesitancy. The vaccination acceptance rate among older adults in Saudi Arabia is low. Interventions designed specifically for older adults addressing worries and concerns related to the vaccine are of paramount importance. In particular, these interventions should be tailored to address gender-based and health literacy level differences.

## 1. Introduction

Many COVID-19 vaccine candidates have been identified as being suitable for emergency use [1]. However, maximizing the adoption of new vaccines that have been created at an extraordinary pace in comparison to past vaccine development timeframes is a serious issue that should be addressed. Vaccine hesitancy is defined as the “delay in acceptance or refusal of vaccination despite the availability of vaccination services” [2]. The World Health Organization (WHO) declared vaccine hesitancy as one of the top 10 global health threats in 2019 [3], as they referred to the rise in anti-vaccination myths by individuals and bots actively spreading fake news, especially on social media [4]. This pre-existing issue was accentuated during the COVID-19 pandemic, and thus poses a barrier to public acceptance of the COVID-19 vaccine, in addition to its overall impact on vaccination reluctance [5].

Since December 2020, COVID-19 vaccines have been introduced across the Kingdom of Saudi Arabia (KSA) in an effort to contain the transmission of the virus [6]. Since then, the focus has shifted to determining the sufficient proportion of the general public that will be willing to be vaccinated and how to ensure they are adequately informed about the vaccine [7]. Vaccination has a direct impact on reducing infection, and vaccination against infectious disease has an indirect impact on herd immunity [8]. In a survey of 2137 Saudi adults, only 48% reported an intention to be vaccinated [9]. Results from a nationally representative study suggest that hesitation and refusal of the vaccine against COVID-19 will hinder the potential to reach herd immunity through immunization [10]. Understanding COVID-19 vaccine acceptance drivers is a global concern, as a lag in vaccination may lead to the rise and spread of variants that can overcome immunity conferred by prior disease and vaccination.

Many factors significantly influence the decision to be vaccinated, including individual risk perception, attitudes, self-efficacy, norms and traditions, barriers, and motivators, as well as social and cultural values [11]. Vaccine uptake among older adults is of particular importance. Due to decreased immunity (immunosenescence) and the high likelihood of existing chronic conditions, older adults are at a greater risk of developing severe illnesses and experiencing potentially serious complications [12,13,14]. Several studies have reported age as a significant risk factor for COVID-19 mortality [15,16]. Age also impacts viral clearance and the duration between hospitalization and death [17,18].

Initially, given the limited availability of the COVID-19 vaccine, expert committees were investigating strategic prioritization plans. Older adults and individuals with comorbidities were designated to be in the first-tier group for vaccine roll-out [19] according to the policy of the Ministry of Health and the Saudi Centre of Disease Prevention and Control. The first phase targeted adults aged above 65 years and the second phase involved adults over 50 years old [6]. This population differs from the general population in a variety of ways, including information processing, risk perception, decision quality, and media consumption [20]. The aim of this study was to investigate the rate of acceptance of the COVID-19 vaccine among older adults (aged 50 and above), identify factors associated with the acceptance of the COVID-19 vaccination, and explore the reasons for hesitancy. Identifying these factors is critical to informing the design of future interventions aimed at maximizing vaccination uptake and effective messaging among this vulnerable group, and to devising effective strategies to support the success of the country’s public health policies.

## 2. Materials and Methods

### 2.1. Study Design and Sample

Data were extracted from an online cross-sectional survey designed to assess the willingness to accept a COVID-19 vaccine in the KSA that was administered from 8 to 14 December 2020 [9,21]. The self-reported questionnaire was designed and developed based on similar studies to assess vaccine acceptance for newly emerging infectious diseases [2,22,23,24]. The questionnaire was initially designed in English and was then translated into Arabic. The Arabic text was used to administer the study. The questionnaire consisted of three main parts. The first part of the questionnaire focused on the respondents’ sociodemographic characteristics. The second part of the questionnaire focused on the respondents’ health status, immunization history, and fear of contracting COVID-19. The third part of the questionnaire focused on the respondent’s willingness to take a COVID-19 vaccine once available.

A simplified-snowball sampling technique was used for recruitment in which participants were asked to send on the invitations to their contacts. The online approach was used to generate valid samples in similar studies conducted in several countries including Saudi Arabia [24,25,26,27]. The survey link included detailed information about the aims and objectives of the study, and the potential to withdraw at any point, along with an assurance of anonymity and confidentiality of the data. Participants were also advised that their participation is voluntary. All adults (≥18 years) living in the KSA were eligible to participate. Once they had provided online informed consent, the participant was directed to the survey. A total of 2137 adults completed the survey out of 2319 originally participated, with a response rate of about 92%. A comprehensive description about the sampling method and data can be found elsewhere [9,21,28]. This study restricted analysis to older adults aged 50 and older, so the main analysis of this study is based on a sample of 488 older adults.

### 2.2. Variables

The main outcome for this study was the acceptance of COVID-19 vaccination among older adults (aged 50 and older). The following statement was provided: “scientists around the world are currently working on a vaccine that could prevent people from being infected with COVID-19. It is hoped that the vaccine will become available in a few months, and the respondents indicated there willingness to receive a COVID-19 vaccine under the scenario that one would be-come available “in the next few months, with an effective rate of the COVID-19 vaccine of 90–95%, provided free by the government”.

To assess the factors that may contribute to vaccine acceptance, information was collected on sociodemographic characteristics: age, gender, marital status, education level, employment status, and income level. Age was stratified as a categorical variable in two groups: 50–59 (the reference category) and ≥60 years. Gender and marital status were also coded as a binary variable (1 = male, 0 = female; 1 = married, 0 = unmarried, including single, widowed, and divorced). Educational attainment was assessed as high school level or below (reference), university degree, and postgraduate degree. Employment categories included government (reference group), private sector, retired, and unemployed. Monthly income (in Saudi Riyal (SR), 1 SR = 0.27 USD) was selected among the following categories: <3000 (reference), 3000–4999, 5000–6999, 7000–9999, 10,000–14,999, 15,000–19,999, 20,000–29,999, and ≥30,000.

Health status was assessed according to the presence or absence of a chronic illness (which has been associated with increased risk of exposure and/or severe COVID-19), prior vaccinated for seasonal influenza, and history of refusal for a physician-recommended vaccine. Psychological factors focused on the degree of perceived risk of COVID-19 infection, scored on a five-point Likert scale (1 = “very low”; 5 = “very high”). This section also assessed the respondents’ personal experience with COVID-19, including family members or friends with current or prior COVID-19 infection, and if any family or friends died from complications associated with COVID-19. Finally, the respondents were asked whether they agree with a mandatory COVID-19 vaccination policy in Saudi Arabia.

### 2.3. Statistical Analyses

The association of categorical variables with the dependent variable (willingness to accept the COVID-19 vaccine) was assessed using bivariate analysis with chi-squared tests. Multivariable logistic regression analysis was used to determine independent predictors of the dependent variable according to the adjusted odds ratio (aOR) and 95% confidence interval (CI). STATA 16.1 (StataCorp LP, College Station, TX, USA) was used for all analyses.

## 3. Results

Among the 488 participants aged 50 and older, 214 (43.85%) stated a willing-ness to accept a free COVID-19 vaccine when available. Table 1 presents the participant characteristics and assessed factors that potentially influence vaccination acceptance among older adults. Among the total participants, 212 (43.44%) were aged ≥ 60 years, 303 (62.09%) were male, 437 (89.55%) were married, 208 (42.62%) had a university degree, around one quarter (26.23%) were employed in the government sector, and 89 (18.24%) had a monthly income ≥30,000 SR.

Table 1 also shows that among the 488 participants, 257 (52.66%) suffered from chronic illness, 255 (52.25%) received the flu vaccine in the past, while 78 (15.98%) refused taking vaccination in the past. Among the participants, 38.32% and 90.57% of their relatives and friends, respectively, were infected by COVID-19. However, 173 (35.45%) participants reported death of a family member or friend from COVID-19. Approximately one-quarter (27.05%) of the respondents were highly concerned about becoming infected with COVID-19 and 29.10% of the participants believed that the vaccine should be mandatory in the KSA. As shown in Table 1, gender, suffering from chronic diseases, history of flu vaccination, history of vaccine refusal, concern about COVID-19 infection, and believing in mandatory vaccination were all significant factors affecting willingness to vaccinate at the 1% level.

Table 2 presents the logistic regression estimates of factors associated with acceptance of a COVID-19 vaccine among older adults in the KSA. Men were more willing to accept vaccination (aOR: 2.277; 95% CI: 1.092–4.745) than women. Moreover, high education levels were associated with greater willingness to be vaccinated, as opposed to those with low level of education. Older adults who had refused vaccination in the past were less likely to be willing to be vaccinated (aOR: 0.358; 95% CI: 0.154–0.830) than those who had not refused vaccination in the past. Those who reported concerns about becoming infected with COVID-19 at a high or very high level were more likely to be willing to be vaccinated (aOR: 4.437; 95% CI: 2.148–9.168) compared with those who had a low or very low level of concerns of becoming infected. Regarding sociodemographic factors, marital status, employment status, and income level had no significant influence on the acceptability of COVID-19 vaccination among older adults in the KSA.

We further investigated the reasons contributing to a lack of willingness to be vaccinated against COVID-19. As shown in Table 3, among the reasons put forward, approximately one-quarter (27.01%) of the respondents indicated that the primary reason for vaccine hesitancy was fear of adverse side effects. This was followed by safety and efficacy concerns (22.63%). The least frequent reason given was the lack of belief of COVID-19 in general (0.36%).

## 4. Discussion

This study complements the emerging picture of COVID-19 vaccine acceptability by focusing on older adults in the KSA. The study puts forth several important key findings to public health authorities engaged in designing vaccine rollout programs or public health communication. The results reveal that the vaccination acceptance rate among the older population in Saudi Arabia reaches 44%. Receiving flu vaccination in the past, high level of risk perception, and believing that COVID-19 vaccination should be mandatory, as well as higher education levels, were found to be predictors of willingness to accept a COVID-19 vaccine. The findings obtained among older adults in this study resemble the findings obtained from a similar study conducted across the general population [9]. Being male, receiving flu vaccination, refusing vaccination in the past, concerns about contracting COVID-19, and believing that COVID-19 vaccination should be mandatory were found to be predictors of willingness to accept COVID-19 vaccine among both older adults and the general population.

Over half of the older adults (56.14%) reported that they are not willing to be vaccinated against COVID-19. In a previous study on the acceptability of a potential COVID-19 vaccine among the general public in Saudi Arabia, only 21% reported a lack of willingness to accept vaccination [29]. In the United States, vaccination hesitancy rates in October 2020 were approximately 50% for participants aged >50 years and 36.2% for older adults aged >60 years. In March 2021, the rates for the same categories fell to 39% and 21%, respectively [30]. The high refusal rate observed in this study could be partly due to the rise of rumors and conspiracy theories about the safety and effectiveness of the vaccine. Such information has the potential to create fear, seed cynicism and doubts over the novel vaccine, consequently threatening public vaccine uptake [31].

The results of this study also offer insights into the individual characteristics that are likely associated with acceptability of the COVID-19 vaccine. Consistent with previous studies, we found that older male participants were more likely to accept vaccination than older female participants [32,33]. This could be explained by gender-based differences in adverse reactions and the humoral immune response following vaccination among women, and greater risks for COVID-19 complications, death, and infectivity among men [34,35,36]. Communication strategies geared at women might therefore be helpful in reducing general reluctance. Moreover, in line with previous studies, we found that vaccine acceptance is higher among highly educated people [37]. Lower educational attainment is linked to lower health literacy, thereby potentially resulting in misinterpretation of COVID-19 vaccination messages [38]. Thus, our findings underline the importance of developing tailored educational campaigns and communication messages that accommodate the differences in health literacy levels among older adults and that are delivered by trusted messengers.

Concerns surrounding the acceptability of a COVID-19 vaccine highlight important aspects for potential educational programs targeted at enhancing vaccination coverage. Major reasons for vaccine refusal among older adults in this study were related to “fear of severe side effects” as well as “safety and efficacy concerns”, which is consistent with prior studies [39,40,41]. In a recent analysis examining 13 studies surrounding vaccine hesitancy, many expressed concerns related to an unknown safety profile [42]. The fear of adverse effects expressed by respondents in this study may reflect the rapid speed of vaccine development and the scarce knowledge about the potential safety of the vaccine at the time of data collection [43]. This suggests that proactive messaging, initiated prior to a large-scale vaccination campaign roll-out, should emphasize the high efficacy rates of COVID-19 vaccines in reducing or eliminating disease, hospitalizations, and death, as well as communicate accurate information about potential side effects, including the rarity of severe adverse events that may have contributed to hesitancy [44,45].

One of the strongest correlates of COVID-19 vaccine acceptability in this study was the participants’ risk perception. Older adults with very high or high risk perceptions were more willing to accept the COVID-19 vaccine. These results coincide with the outcome of earlier studies showing correlations between perceived risk of infection and acceptability of the COVID-19 vaccine [24]. Participants in this study who believed that the COVID-19 vaccine should be made mandatory were more likely to be willing to accept vaccination for COVID-19. Although mandatory vaccination was not ruled out in Saudi Arabia at the time of data collection, mandatory vaccination can be warranted when confidence in safety and effectiveness is high, the expected utility of compulsory vaccination is higher than the alternatives, costs or penalties for non-compliance are proportionate, and if the threat to public health is critical [46]. Furthermore, in line with previous studies across different countries [40,47,48] and diseases [49,50], the results of this study show that previous vaccination behavior is a strong predictor of vaccine acceptability. Concerns related to the need for further information about the safety and utility of the vaccine have been reported to be a key reason for past vaccine refusal [40]. Moreover, previous studies have shown that vaccine acceptance is an individual habit that might apply to different diseases sharing the same mode of transmission and clinical characteristics [51,52].

This study is not without limitations. Although a high number of older adults use online applications and communication channels, the online nature of this survey may have limited the access to a larger number of potential participants. Moreover, participants were recruited by convenience sampling; thus, caution should be taken when applying the results of this study to other settings or populations. Furthermore, the self-administered questionnaire may be subject to social desirability. A further limitation is the cross-sectional design of this study and the lack of available data on non-responders. Finally, the online survey used in this study could have impacted the generalizability of our results.

## 5. Conclusions

This study offers insight into the emerging global picture of the acceptability of the COVID-19 vaccine by primarily focusing on older adults in the KSA. Given the low level of vaccination acceptance among older adults, continuous encouragement to accept the COVID-19 vaccine among the vulnerable could result in reduced morbidity and mortality. Attention and efforts considering gender-based and educational attainment differences are needed in promoting vaccine acceptability. Effective communication of information that emphasizes the safety and efficacy aspects of these novel vaccines is necessary in order for vaccination to be successful among the older population.

## Figures and Tables

**Table 1:** Frequency distribution and chi-squared analysis of intentions of COVID-19 vaccination among the older adults (*n* = 488).

Variable	No Intention to Receive COVID-19 Vaccine (*n* = 274)		Intention to Receive COVID-19 Vaccine (*n* = 214)		Total (*n* = 488)		*p*-Value
	*n*	%	*n*	%	*n*	%	
**Age (years)**							
50 to 59	156	56.93	120	56.07	276	56.56	0.849
≥60	118	43.07	94	43.93	212	43.44	
**Gender**							
Female	119	43.43	66	30.48	185	37.91	<0.001 ***
Male	155	56.57	148	69.16	303	62.09	
**Marital Status**							
Unmarried	31	11.31	20	9.35	51	10.45	0.481
Married	243	88.69	194	90.65	437	89.55	
**Educational attainment**							
High school or below	71	25.91	51	23.83	122	25.00	0.646
University degree	119	43.43	89	41.59	208	42.62	
Postgraduate degree	84	30.66	74	34.58	158	32.38	
**Employment status**							
Government employee	65	23.72	63	29.44	128	26.23	0.270
Private sector employee	39	14.23	20	9.35	59	12.09	
Unemployed	36	13.14	29	13.55	65	13.32	
Retired	134	48.91	102	47.66	236	48.36	
**Monthly Income (in SR)**							
˂3000	16	5.84	12	5.61	28	5.74	0.582
3000–4999	9	3.28	14	6.54	23	4.71	
5000–6999	16	5.84	10	4.6	26	5.33	
7000–9999	26	9.49	19	8.88	45	9.22	
10,000–14,999	56	20.44	37	17.29	93	19.06	
15,000–19,999	60	21.90	44	20.56	104	21.31	
20,000–29,999	47	17.15	33	15.42	80	16.39	
≥30,000	44	16.06	45	21.03	89	18.24	
**Health status**							
No chronic disease	145	52.92	86	40.19	231	47.34	<0.001 ***
Chronic disease diagnosis	129	47.08	128	59.81	257	52.66	
**Vaccination history**							
Never received flu vaccine	158	57.66	75	35.05	233	47.75	<0.001 ***
Have received flu vaccine	116	42.34	139	64.95	255	52.25	
**Vaccine willingness history**							
Never refused recommended vaccine	210	76.64	200	93.46	410	84.02	<0.001 ***
Have refused recommended vaccine	64	23.36	14	6.54	78	15.98	
**Family member tested positive for COVID-19**							
No	161	58.76	140	65.42	301	61.68	0.133
Yes	113	41.24	74	34.58	187	38.32	
**Friend tested positive for COVID-19**							
No	24	8.76	22	10.28	46	9.43	0.568
Yes	250	91.24	192	89.72	422	90.57	
**Death of friend/family from COVID-19**							
No	181	66.06	134	62.62	315	64.55	0.430
Yes	93	33.94	80	37.38	173	35.45	
**Perceived risk of becoming infected with COVID-19**							
Low or very low	136	49.64	51	23.83	187	38.32	<0.001 ***
Fair	79	28.83	90	42.06	169	34.63	
High or very high	59	21.53	73	34.11	132	27.05	
**Support mandatory COVID-19 vaccination in Saudi Arabia**							
No	268	97.81	78	36.45	346	70.90	<0.001 ***
Yes	6	2.19	136	63.55	142	29.10	

*** *p* < 0.01.

**Table 2:** Logistic regression estimates of factors associated with acceptance of COVID-19 vaccination among the older adults (*n* = 488).

Variable	aOR	95% CI	*p*-Value
**Age (years)**			
50 to 59 (ref)			
≥60	1.258	0.688–2.300	0.456
**Gender**			
Female (ref)			
Male	2.277	1.092–4.745	0.028 **
**Marital Status**			
Unmarried (ref)			
Married	0.632	0.260–1.535	0.311
**Educational attainment**			
High school or below (ref)			
University degree	1.896	0.880–4.084	0.090 *
Postgraduate degree	3.040	1.270–7.277	0.013 **
**Employment status**			
Government employee (ref)			
Private sector employee	0.449	0.165–1.226	0.118
Unemployed	2.397	0.793–7.242	0.121
Retired	0.957	0.457–2.004	0.908
**Monthly Income (in SR)**			
˂3000			
3000–4999	2.728	0.395–18.856	0.309
5000–6999	1.963	0.307–12.565	0.477
7000–9999	3.169	0.604–16.619	0.172
10,000–14,999	1.736	0.350–8.623	0.500
15,000–19,999	1.582	0.316–7.914	0.577
20,000–29,999	1.285	0.248–6.668	0.765
≥30,000	2.560	0.502–13.052	0.258
**Health status**			
No chronic disease			
Chronic disease diagnosis	1.169	0.668–2.044	0.585
**Vaccination history**			
Never received flu vaccine			
Have received flu vaccine	1.661	0.959–2.878	0.070 *
**Vaccine willingness history**			
Never refused recommended vaccine			
Have refused recommended vaccine	0.358	0.154–0.830	0.017 **
**Family member tested positive for COVID-19**			
No (ref)			
Yes	1.098	0.612–1.969	0.755
**Friend tested positive for COVID-19**			
No (ref)			
Yes	1.132	0.406–3.158	0.812
**Death of friend/family from COVID-19**			
No (ref)			
Yes	1.035	0.589–1.818	0.905
**Perceived risk of becoming infected with COVID-19**			
Low or very low (ref)			
Fair	3.473	1.780–6.777	<0.001 ***
High or very high	4.437	2.148–9.168	<0.001 ***
**Support mandatory COVID-19 vaccination in Saudi Arabia**			
No (ref)			
Yes	101.761	39.173–264.348	<0.001 ***

*** *p* < 0.01, ** *p* < 0.05, * *p* < 0.1.

**Table 3:** Reasons for vaccine hesitancy among older adults.

Reason	*n*	%
Fear of adverse side effects	74	27.01
Safety and efficacy concerns	62	22.63
The speed of making the vaccine	11	4.01
The short duration of clinical trials	29	10.58
Personal desire not to be vaccinated	20	7.30
I think the vaccine is a plot	31	11.31
I do not believe in the existence of COVID-19	1	0.36
I feel that masks and sanitizers are sufficient for protection	23	8.39
Other	23	8.39
Total	274	100

## Data Availability

The datasets generated and/or analyzed during the current study are not publicly available due to privacy and confidentiality agreements as well as other restrictions but are available from the corresponding author on reasonable request.
